# Avoidance of Apoptosis in Embryonic Cells of the Annual Killifish *Austrofundulus* *limnaeus* Exposed to Anoxia

**DOI:** 10.1371/journal.pone.0075837

**Published:** 2013-09-18

**Authors:** Camie L. Meller, Jason E. Podrabsky

**Affiliations:** Department of Biology, Portland State University, Portland, Oregon, United States of America; Leibniz Institute for Age Research - Fritz Lipmann Institute (FLI), Germany

## Abstract

Embryos of the annual killifish *Austrofundulus* *limnaeus* have unequalled ability among vertebrates to survive long-term anoxia. Surprisingly, these embryos can survive for months in anoxia despite a large-scale decrease in ATP levels during the initial hours of anoxic exposure. These conditions are known to trigger apoptotic cell death in mammalian cells as a result of ischemia or anoxia. Anoxia-induced induction of apoptosis was investigated in four developmental stages of *A* *. limnaeus* that differ in their tolerance of anoxia, and thus may respond to anoxia uniquely. Exposure to staurosporine was used to determine if *A* *. limnaeus* cells were competent to enter apoptosis via cues other than anoxia. Apoptotic cells were identified by TUNEL assays and by measuring caspase 3/7 activity. Exposure to 48 hr of anoxia did not induce an increase in TUNEL-positive cells and generally did not lead to an increase in caspase 3/7 activity. However, treatment of anoxic embryos with 10 μM staurosporine resulted in a significant increase in caspase 3/7 activity in both normoxic and anoxic embryos. These results suggest that apoptosis is avoided in embryos of *A* *. limnaeus* following exposure to anoxia at least in part by mechanisms that prevent the activation of caspase 3/7 activity. While this mechanism remains unknown, it may be triggered by a protein kinase that can be experimentally inhibited by staurosporine.

## Introduction

Anoxia is a powerful cellular stressor that induces necrotic or apoptotic cell death within minutes in mammalian cells [1]. Inhibition of mitochondrial metabolism due to a lack of oxygen quickly leads to exhaustion of cellular energy stores in the form of creatine phosphate and ATP, dissipation of ion gradients across cellular membranes, and the permeabilization of the mitochondrial membranes to key ions and proteins that can trigger events leading to apoptotic cell death [1-4]. In contrast to mammals, embryos of the annual killifish *Austrofundulus* *limnaeus* can survive for months at 25°C in the complete absence of oxygen [5,6]. Despite this incredible ability, very little is known about the cellular mechanisms that support survival of anoxia in this species.

*Austrofundulus* *limnaeus* is an annual killifish that lives in ephemeral ponds in the Maracaibo basin of Venezuela [7]. Like other annual killifish, *A* *. limnaeus* produces embryos that can enter into a profound state of metabolic depression termed diapause at three distinct stages of development, diapause I, II and III [8-10]. Diapause I may occur early in development at the end of epiboly but prior to the formation of the neural keel [8]. Diapause II occurs about midway through development at the completion of somitogenesis and just prior to organogenesis in an embryo that features a differentiated central nervous system, a functional tubular heart, and 38-42 pairs of somites [8]. Diapause III occurs at the completion of embryonic development in an embryo that has consumed most of the yolk resources and is ready to feed almost immediately upon hatching [8].

Tolerance of long-term anoxia in embryos of *A* *. limnaeus* is gained during early development and peaks with a lethal time to 50% mortality (LT_50_) of around 65 days when embryos enter diapause II [5,6]. This extreme tolerance of anoxia is retained for at least 4 days of post-diapause II development, but then is slowly lost as embryos develop towards diapause III [5,6]. Anaerobic metabolism in anoxia tolerant embryos is supported by accumulation of lactate, succinate, and large amounts of γ-aminobutyric acid (GABA) [6]. Post-diapause II embryos respond to anoxia by entering a profound state of metabolic depression with heart rates dropping from over 80 to 0 beats per minute [11] and rates of heat dissipation decreasing to those exhibited by dormant diapause II embryos [12]. In both diapause II embryos and those at 4 days post-diapause II, ATP levels plummet during the initial hours of exposure to anoxia to as low as 20% of normoxic levels [12]. Loss of ATP leads to significant increases in AMP and a reduction in adenylate energy charge (an index of cellular energetic status) from near 1.0 to below 0.4 [12]. The cellular consequences of this drastic reduction in ATP levels and indicators of cellular energy status are currently unknown in embryos of *A* *. limnaeus* , but would almost certainly lead to cell death in most mammalian cells.

Cell death is a necessary and vital feature of normal development and organismal function [13]. However, ischemic or anoxic stress in mammalian cells can activate cell death pathways inappropriately leading to increases in tissue damage and ultimately compromising tissue function and organismal survival. Thus, inappropriate stress-induced activation of apoptotic pathways during development could have drastic results that lead to abnormal development or even developmental failure. In this study we report for the first time that cells of developing *A* *. limnaeus* embryos are competent to enter apoptosis, but that exposure to anoxia does not activate this cell death pathway.

## Materials and Methods

### Ethics statement

All procedures involving animals were performed according to the Guide for the Care and Use of Laboratory Animals of the National Institutes of Health under a protocol approved by the PSU Institutional Animal Care and Use Committee (approval number psu12.03.22.2).

### Husbandry and Collection of embryos

Adult *Austrofundulus* *limnaeus* were cared for as described previously [14]. Spawning pairs of fish were housed in 9.5 l glass aquaria with 21 tanks connected to a common sump and a total system volume of 190 l. Water temperature was regulated at 26-28°C. Water was changed (10% of system volume) twice daily. Fish were fed twice daily during the workweek and daily on the weekends with frozen bloodworms (chironomid larvae, Hikari) or chopped live earthworms. Embryos were collected through natural spawning activity twice weekly as described previously [14]. Embryos were incubated in 100 x 15 mm plastic Petri dishes at a density of 100 embryos per dish for early development through diapause II, and 50 embryos per dish during post-diapause II development. Embryo incubation medium was composed of dilute salts (1 ppt salinity) that mimic the natural waters inhabited by *A* *. limnaeus* [14]. For the first 4 d of development methylene blue was added to the medium to reduce bacterial and fungal growth. At 4 days post-fertilization (dpf) embryos were subjected to a dilute hypochlorite bleaching protocol to reduce microbial growth [14]. Following the bleaching protocol embryos were transferred to embryo medium containing 10 μg/l gentamycin for the duration of development. Embryos were incubated at 25°C in the dark in constant temperature incubators (Sheldon Manufacturing, Cornelius, OR) unless otherwise noted. For post-diapause II embryos, diapause II was broken experimentally by exposing embryos to a long day photoperiod (14 hr light, 10 hr dark) at 30°C for 48 hr at which time they were returned to incubation at 25°C in the dark. A large percentage of the embryos break diapause II within 2-3 d after this treatment. Embryos were inspected daily following the treatment and sorted into synchronously developing groups of post-diapause II embryos.

Four different embryonic stages were chosen for study (Table 1): actively developing pre-diapause II embryos at 16 dpf, diapause II embryos at 32 dpf, actively developing post-diapause II embryos with extreme anoxia tolerance at 4 days post-diapause II (dpd), and actively developing post-diapause II embryos at 12 dpd which have a greatly reduced tolerance of anoxia but respond to anoxic preconditioning [15]. Preconditioning is a phenomenon where a brief exposure to anoxia/ischemia induces endogenous protective mechanisms that reduce damage and improve survival of subsequent harmful bouts of anoxia or ischemia [16]. Three to five independent sample sets (spawning events) were prepared for each of the four developmental time points. A matched normoxic control sample was prepared for each set of anoxic samples.

**Table 1:** Stages of development investigated.

dpf^1^	dpd^2^	WS^3^	Stage	Anoxia LT_50_^4^	Precond^5^
16			Mid-somitogenesis	40	no data
32	0	32	Diapause II	65	no
	4	36	Early organogenesis	75	no
	12	40	Late organogenesis	6	yes

^1^ days post-fertilization

^2^ days post-diapause II

^3^ Wourms’ stage [9]

^4^ Lethal time to 50% mortality in days at 25°C [5,6].

^5^ Ability to respond to anoxic preconditioning.

### Exposure to anoxia

Whole embryos were exposed to anoxia in a Bactron III anaerobic chamber (Sheldon Manufacturing, Cornelius, OR) that uses positive pressure to maintain an atmosphere of 5% H_2_, 5% CO_2_, and 90% N_2_ gas. Residual oxygen is removed from the chamber through the formation of water from hydrogen and oxygen via the action of a palladium catalyst. Embryos were sampled after 48 hr of anoxia and following 1, 4, 24, and sometimes 72 hr of aerobic recovery from anoxia at each stage of development indicated in Table 1. All embryonic stages were followed for 24 hr of aerobic recovery while post-diapause II stages were also sampled after 72 hr of aerobic recovery.

### Staurosporine exposures

Staurosporine is widely used to induce apoptosis in animal cells [17-20] and was used in this study to test the competence of *A* *. limnaeus* cells to undergo apoptotic cell death. Both whole embryos and dissociated cells isolated from embryos were investigated. Dissociated cells from 8 dpf embryos were treated for 48 hr with 1 μM staurosporine (Sigma Chemical) in L-15 medium supplemented with 1% DMSO, 50 U/ml penicillin, 50 μg/l streptomycin, and 50 μg/l gentamycin. Control cells were treated as above without the staurosporine. Three independent cell isolations were used to replicate each exposure (n=3). Whole embryos were treated with 10 μM staurosporine from 9-11 dpf in embryo medium supplemented with 10 μg/l gentamycin and 1% DMSO to improve delivery of the staurosporine. Control embryos were treated as above without the staurosporine. Three groups of embryos were exposed separately to a time-course of exposure to staurosporine (n=3).

### TUNEL assay

Apoptotic cells were identified using terminal deoxynucleotidyl transferase dUTP nick end labeling (TUNEL) to detect DNA fragmentation. TUNEL assays were performed on isolated cells collected from approximately 50 to 100 embryos dissociated into cell suspensions by mechanical dissociation with a glass pestle through a 24 mm, 500 µm-mesh basket (Netwell™, Corning Product #3480) into a 50 mm plastic Petri dish containing 2 ml of phosphate buffered saline (PBS). Cell suspensions were then transferred to a 1.7 ml microcentrifuge tube and pelleted by centrifugation (300 x *g*, 5 min). The cells were then rinsed once in PBS and resuspended in 100 μl of PBS, and fixed overnight at room temperature by the addition of 1 ml of 3.7% paraformaldehyde (final concentration of 3.4% paraformaldehyde). The cells were then pelleted as above and rinsed once in 1 ml of PBS, pelleted and resuspended in final volume of 100 μl of PBS. Cells were permeabilized by the addition of 1 ml of 100% methanol (final concentration of 90% methanol) and stored at -20°C until the day of the assay.

A TUNEL assay kit from Phoenix Flow Systems (APO-BRDU, *AU-1001*) was used according to the manufacturer’s instructions to quantify TUNEL-positive cells. On the day of the assay, the methanol-fixed cells were pelleted via centrifugation (300 x *g* for 5 min) and rinsed once in wash buffer (Phoenix Flow Systems). The cell pellet was resuspended in 50 μL of DNA labeling solution and incubated at 37°C for 1 hr with shaking, followed by static overnight incubation at room temperature. Cells were then rinsed and resuspended in 100μl of anti-Br-dUTP antibody solution followed by incubation for 30 min in the dark at room temperature. After the antibody incubation, 0.5 ml of propidium iodide/RNase A solution (Phoenix Flow Systems) was added to each sample for 30 min in the dark at room temperature. All samples were analyzed within 3 hr of staining. Positive- and negative-control cells provided in the kit (Phoenix Flow Systems) were used to ensure that the kit reagents and equipment were working properly. A species-specific positive control was generated by treating dissociated *A* *. limnaeus* embryonic cells at 8 dpf with 1 µM staurosporine in L-15 medium for 24 hr (see above).

An inverted fluorescence microscope (Leica, DMIRB) equipped with filter sets to capture both green (TUNEL) and red (nuclear DNA stained with propidium iodide) fluorescence was used to visualize and quantify stained cells. For each microscopic field of view [400X] images were captured using a Leica DC480 cooled CCD camera at 623 nm for propidium iodide (PI) fluorescence and at 520 nm for FITC fluorescence. For each sample, multiple fields of view were used to quantify the number of cells that were TUNEL-positive as well as the total number of PI-positive cells.

### Caspase 3/7 Activity

Caspase-3/7 activity was measured in freshly dissociated cells isolated from embryos following experimental treatments. Whole embryos were exposed to anoxia as described above and then placed on ice prior to cell dissociation as described above for TUNEL assays with the exception that dissociated cells were collected in 1 ml of L-15 cell culture medium. Cell suspensions were pelleted by centrifugation (300 x *g*, 5 min) and rinsed once in L-15 medium followed by resuspension in 1 ml of fresh L-15. Ten µl of each concentrated cell suspension was used to calculate the cell density using a disposable counting chamber (C-Chip # DHC-N01). Each cell suspension was diluted to 150,000 cells/100 µl and 75,000 cells were used for each assay.

A Caspase-Glo® 3/7 Assay kit (Promega, #G8091) that relies on caspase-dependent cleavage of a DEVD tetrapeptide sequence was used to measure caspase activity in the cell suspensions. Caspase reagent (50 µl) was added to 50 µl of cell suspension and incubated at room temperature for 1 hr. A reagent “blank” was prepared each day using 50 µl of L-15 medium and 50 µl of caspase reagent. Positive controls were created by harvesting cells from 9 dpf *A* *. limnaeus* embryos exposed to 10 µM staurosporine for 24 hr under normoxic or anoxic incubation (see above). Luminescence was measured using a luminometer (Kikkoman model C-110). Triplicate readings were taken of each sample and averaged.

### Data presentation and statistics

Graphs were prepared and statistical analyses were performed using GraphPad Prism 5.0 software. Statistical significance was set at p < 0.05 for all comparisons. When appropriate Student Newman-Keuls post-hoc comparison test was used to compare individual means. Percentage data were transformed using the arcsine of the square root of the proportion transformation prior to statistical analysis.

## Results

### TUNEL assay

**Figure 1:**
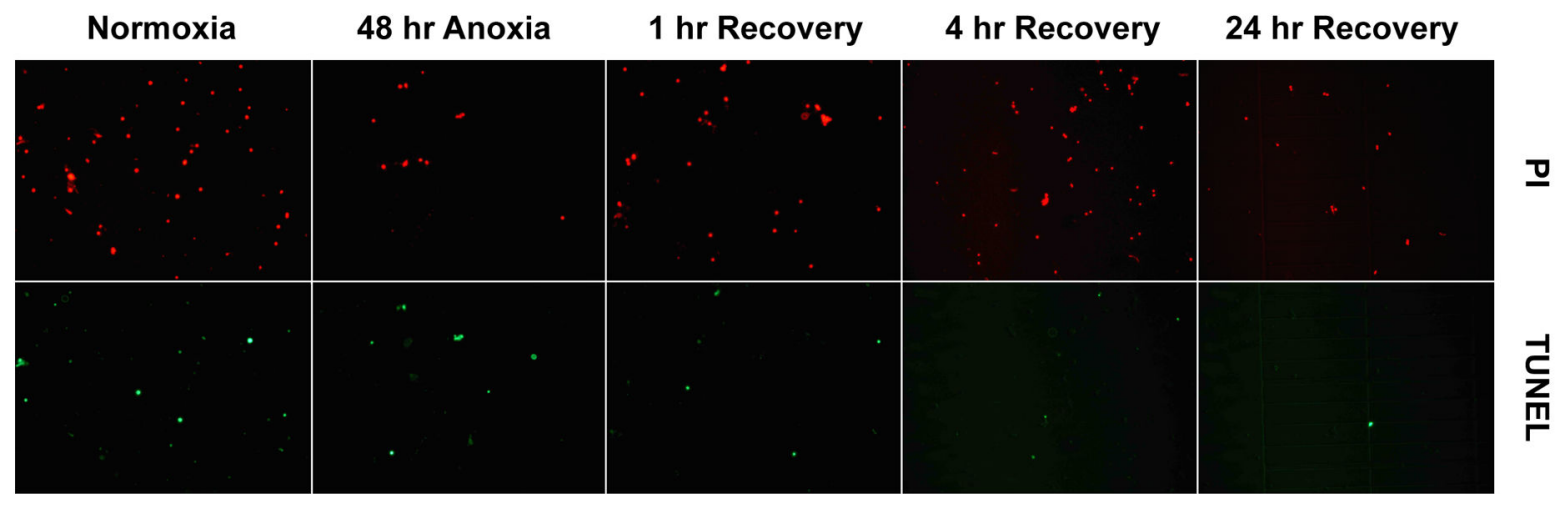
Representative images of TUNEL assays. Microscope fields at 400x magnification used to quantify total number of cells as indicated by propidium iodide (PI) staining of nuclei and TUNEL-positive (potentially apoptotic) cells. These images are from 16 dpf embryos.

**Figure 2:**
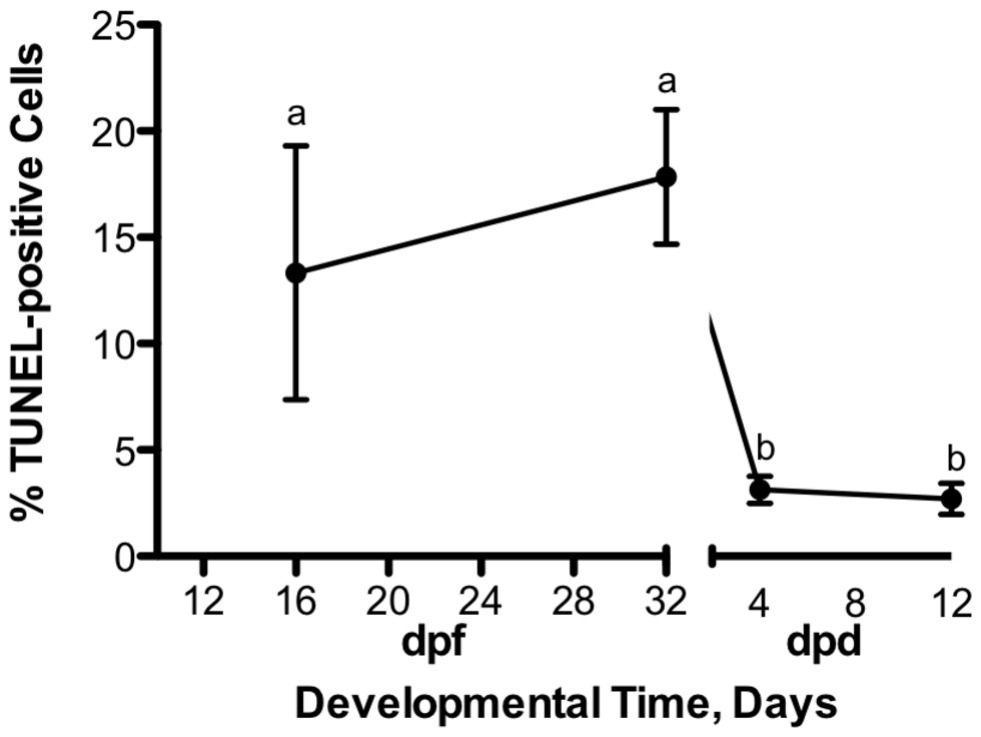
Developmental patterns of TUNEL-positive cells in normoxic embryos of *A. limnaeus*. Embryos at 16 dpf and during diapause II have significantly higher proportions of TUNEL-positive cells compared to post-diapause II embryos. Symbols with different letters are statistically different (Student Newman-Keuls, p < 0.05). The x-axis represents developmental time in days post-fertilization (dpf) for early embryos and days post-diapause II (dpd) for post-diapause II embryos. Symbols represent means ± S.E.M. (n = 3-5).

**Figure 3:**
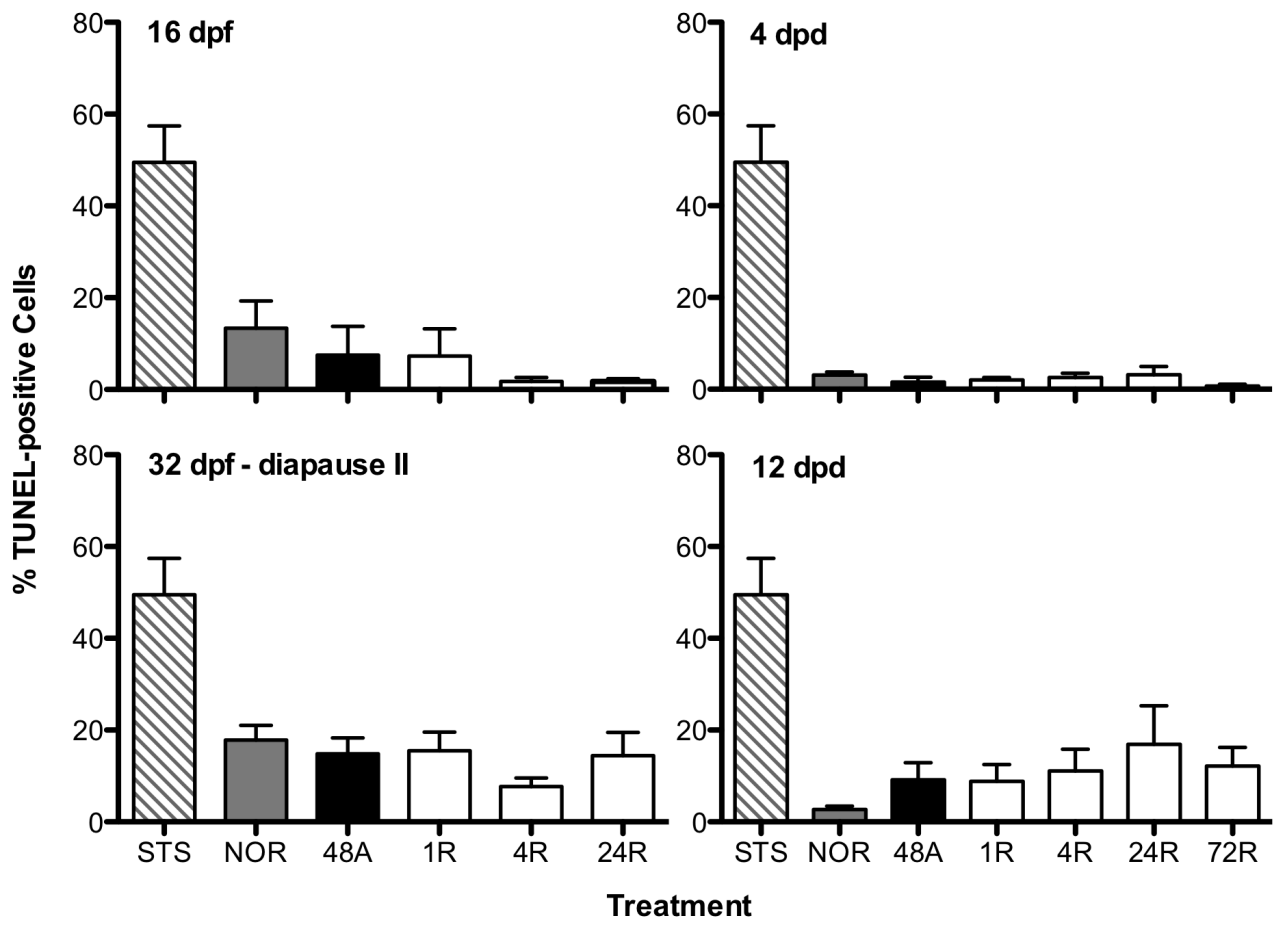
Anoxia does not alter the proportion of TUNEL-positive cells in embryos of *A. limnaeus.* No difference in the proportion of TUNEL-positive cells was observed in embryos following exposure to anoxia (ANOVA, p > 0.05). Treatments are listed on the x-axis as follows: STS = isolated cells from 8 dpf embryos treated with 1 μM staurosporine for 24 hr; NOR = normoxic embryos at t = 0; 48A = 48 hr of anoxia; 1R = 1 hr of aerobic recovery; 4R = 4 hr of aerobic recovery; 24R = 24 hr of aerobic recovery; 72R = 72 hr of aerobic recovery. Bars represent the means ± S.E.M. (n = 3-5).

Typical TUNEL-assay results are presented in Figure 1. Under normoxic conditions, early embryos (16dpf and diapause II) have a significantly higher proportion of TUNEL-positive cells (ANOVA, p = 0.003) compared to the later post-diapause II stages (Figure 2). Exposure to anoxia had no statistically significant effect on the proportion of TUNEL-positive cells observed within any of the developmental stages investigated (Figure 3). In fact, in some instances the trend was for a decrease in TUNEL-positive cells following 48 hr of anoxia. The only exception to this pattern is in the late developing embryos at 12dpd, which showed an overall increase in TUNEL-positive cells, although this effect is not statistically significant (ANOVA, p > 0.05).

### Caspase 3/7 Activity

**Figure 4:**
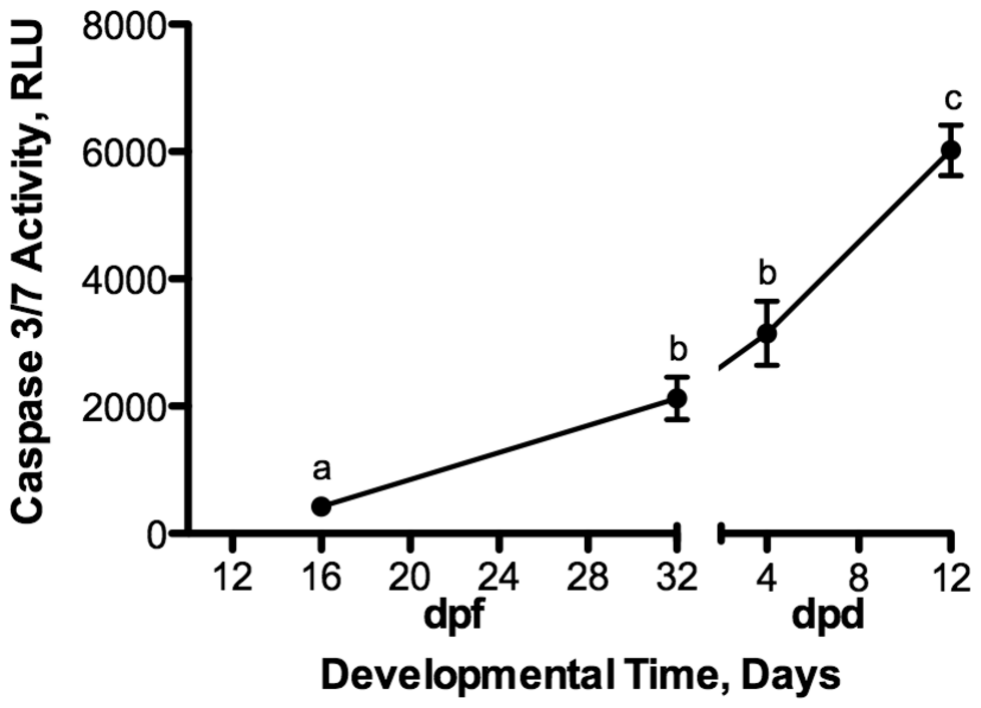
Caspase 3/7 activity increases during normoxic development in embryos of *A. limnaeus.* Activity is expressed in relative luminescence units (RLU). Symbols with different letters are statistically different (Student Newman-Keuls, p < 0.05). The x-axis represents developmental time in days post-fertilization (dpf) for early embryos and days post-diapause II (dpd) for post-diapause II embryos. Symbols represent means ± S.E.M. (n = 3-4).

**Figure 5:**
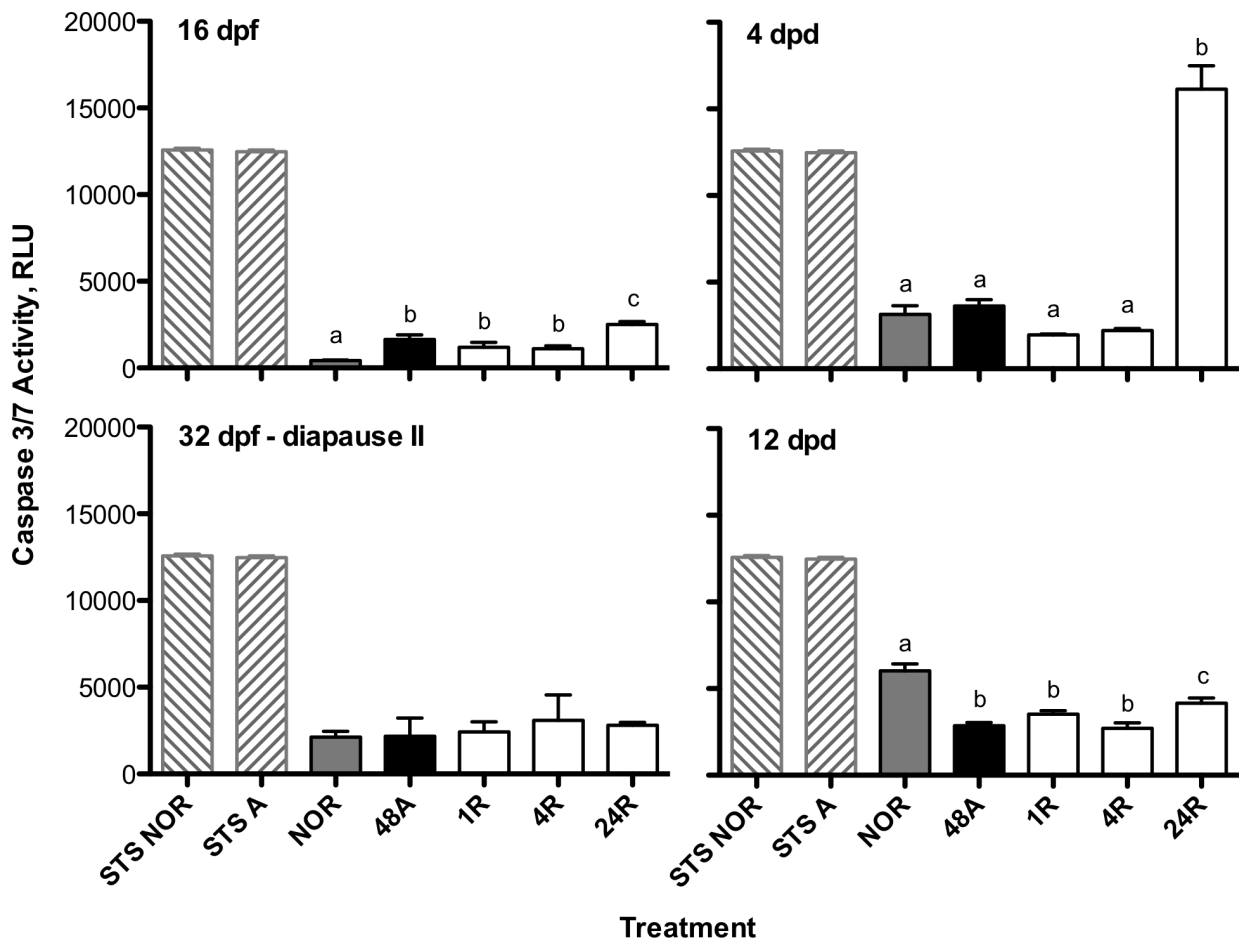
Caspase 3/7 activity responds to anoxia in a stage-specific manner in embryos of *A. limnaeus.* Activity is expressed in relative luminescence units (RLU). Treatments are listed on the x-axis as follows: STS NOR = cells isolated from normoxic 9 dpf embryos treated for 24 hr with 10 μM staurosporine; STS A = cells isolated from anoxic 9 dpf embryos treated for 24 hr with 10 μM staurosporine; NOR = normoxic embryos at t = 0; 48A = 48 hr of anoxia; 1R = 1 hr of aerobic recovery; 4R = 4 hr of aerobic recovery; 24R = 24 hr of aerobic recovery. Bars represent the means ± S.E.M. (n = 3-5). Within each developmental stage, bars with different letters are statistically different (Student Newman-Keuls, p < 0.05).

Caspase activity under normoxic conditions increased significantly (ANOVA, p < 0.0001) as a function of developmental progression (Figure 4). Overall, caspase levels are low in embryos of *A* *. limnaeus* regardless of treatment or developmental stage when compared to the embryos treated with staurosporine (Figure 5). However, there are some stage-specific responses to anoxia in caspase 3/7 activity that are worthy of note. Early embryos at 16 dpf exhibit a statistically significant increase in caspase 3/7 activity in response to anoxia (Figure 5; ANOVA, p < 0.0001), although these increases are very small compared to embryos treated with staurosporine. Diapause II embryos respond to anoxia with no detectable change in caspase 3/7 activity. Post-diapause II embryos at 4 dpd experience a significant increase in activity after 24 hr of aerobic recovery (ANOVA, p < 0.0001) while embryos at 12 dpd expressed a significant decrease in caspase 3/7 activity in response to anoxia and during recovery from anoxia (ANOVA, p < 0.0001; Figure 5).

**Figure 6:**
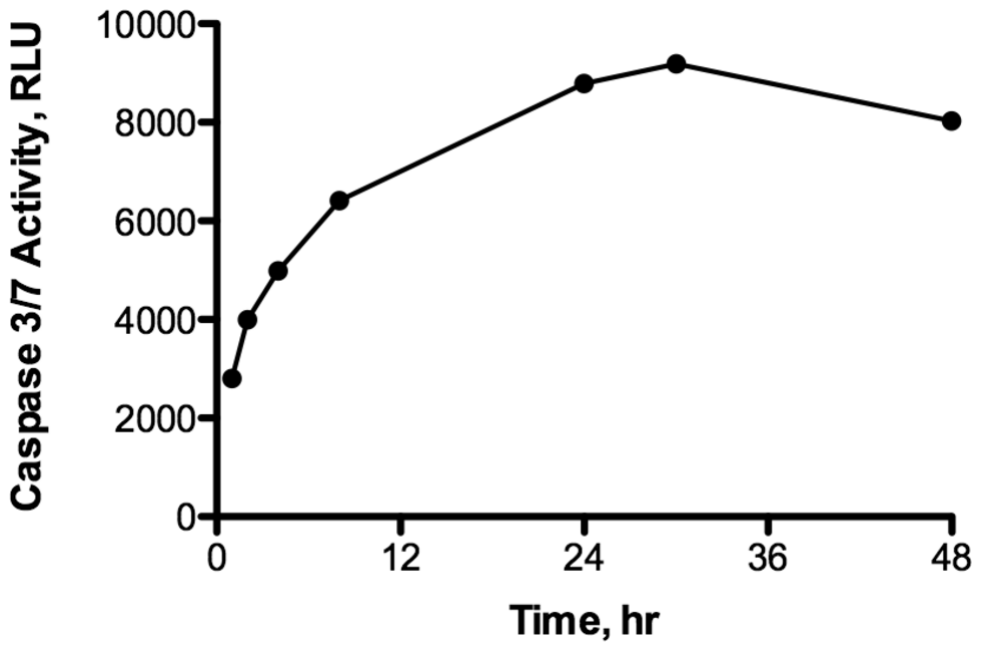
Staurosporine induces caspase 3/7 activity in cells isolated from 8 dpf embryos of *A. limnaeus.* Activity is expressed in relative luminescence units (RLU). Induction of caspase activity is statistically detectable after only 1 hr and peaks at 30 hr (ANOVA, Student Newman-Keuls p < 0.001). Symbols represent means ± S.E.M, (n=3; error bars are within symbols).

**Figure 7:**
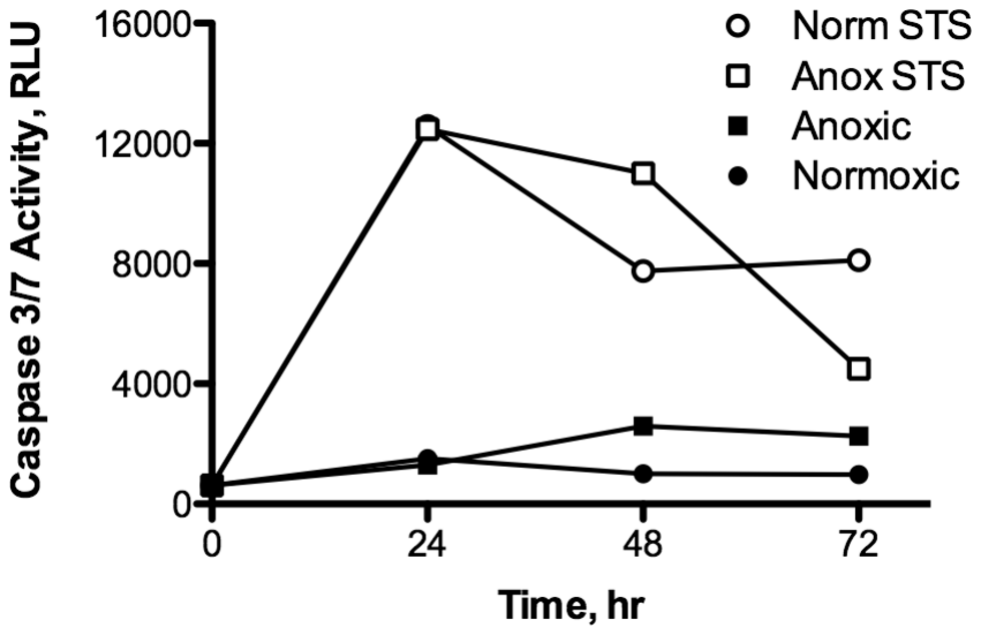
Staurosporine induces caspase 3/7 activity in anoxic and normoxic embryos of *A. limnaeus.* Activity is expressed in relative luminescence units (RLU). Caspase activity peaks at 24 hr (ANOVA, Student Newman-Keuls p < 0.001). Symbols represent means ± S.E.M (n=3; error bars are within symbols). STS = staurosporine treated.

Staurosporine (1 μm) was able to induce caspase 3/7 activity in isolated cells of *A* *. limnaeus* (Figure 6). Induction was rapid with the 1 hr time-point already statistically different from control levels (ANOVA, Student Newman-Keuls, p < 0.001). Caspase activity peaked after about 30 hr of continuous treatment. Whole embryos exposed to 10 μM staurosporine also responded with a significant increase in caspase 3/7 activity (Figure 7, ANOVA, Student Newman-Keuls, p < 0.001). Interestingly, both normoxic and anoxic embryos experienced a similar increase in caspase activity in response to staurosporine that peaked after 24 hr of continuous exposure. Treatment with 1% DMSO alone had very little effect on caspase 3/7 activity as illustrated by both normoxic and anoxic control embryos not exposed to staurosporine (Figure 7).

## Discussion

### Apoptosis is avoided in response to 48 hr of anoxia

Cells of *A* *. limnaeus* embryos appear to be quite resistant to anoxia-induced apoptosis as evidenced by a complete lack of increase (and in fact a trend for a decrease) in TUNEL-positive cells and caspase 3/7 activity following 48 hr of anoxia. One interesting result of this work is the high proportion of TUNEL-positive cells in early embryos (16 dpf and diapause II) compared to post-diapause II embryos. Although generally considered a good indicator of apoptosis, positive TUNEL staining may also be associated with gene transcription [21], DNA repair [22], and non-apoptotic DNA damage [23,24]. It is interesting that a high proportion of TUNEL-positive, but likely not apoptotic cells were also reported for hibernating but not summer active ground squirrel gut epithelial cells [24]. Perhaps there is some change in DNA/chromatin structure or nuclear RNA levels associated with metabolic dormancy that is causing TUNEL-positive staining in the absence of apoptosis. Due to the consistently low numbers of TUNEL-positive cells in normoxic post-diapause II embryos, and a clear lack of increase in TUNEL-positive cells and low caspase 3/7 activity, we conclude that the high levels of TUNEL-positive cells in 16 dpf and diapause II embryos under normoxic conditions are probably due to some factor other than apoptosis or cell death.

### Stage-specific response of caspase 3/7 activity to anoxia

Caspase 3 and 7 are the major executioner caspases in vertebrate cells and are typically activated by both the intrinsic (mitochondrial) and extrinsic pathways for induction of apoptosis [25,26]. Thus, significant increases in caspase-3/7-like activity would be consistent with activation of apoptotic cell death.

Early embryos at 16 dpf respond to exposure to anoxia with a modest increase in caspase 3/7 activity that peaks after 24 hr of aerobic recovery. However, this increase is small in comparison to the activity associated with exposure to staurosporine, and clearly does not lead to an increase in TUNEL-positive cells after 24 hr of aerobic recovery. Thus, we conclude that the modest increase in activity is either not sufficient to induce an apoptotic response or that embryos express caspase inhibitors [27] that are blocking the proteolytic action of caspase 3/7 activity (see discussion below). Although diapause II embryos express higher normoxic levels of caspase 3/7 activity than 16 dpf embryos, they lack a response in caspase activity to anoxia or recovery from anoxia. This lack of induction may be a mechanism for preventing activation of apoptotic pathways during metabolic dormancy when energy flow is extremely low.

Post-diapause II embryos at 4 dpd experience a significant increase in caspase 3/7 activity after 24 hr of aerobic recovery from 48 hr of anoxia. However, apoptosis is not induced in these embryos as evidenced by extremely low levels of TUNEL-positive cells at 24 and 72 hr of aerobic recovery. In contrast, embryos at 12 dpd exhibit a significant decrease in caspase 3/7 activity in response to anoxia. A higher basal level of apoptosis in 12 dpd embryos that is actively inhibited during exposure to anoxia may explain this pattern.

### Staurosporine effects on caspase 3/7 activity

One very interesting result of this study is the induction of caspase 3/7 activity by staurosporine in embryos exposed to anoxia. This observation suggests that caspase 3/7 activity is actively blocked in anoxia treated embryos by a mechanism that is disrupted by staurosporine. Staurosporine is a potent (pM to nM range) and rather nonspecific inhibitor of protein kinases [28]. Thus, these data suggest an active role for protein kinases in blocking an increase in caspase 3/7 activity in response to anoxia. Unfortunately, TUNEL assays were not performed on these embryos, and thus it is presently not clear if these embryos would have exhibited an apoptotic phenotype. Future studies will be required to clarify this point, but the present study suggests a role for a staurosporine-sensitive kinase in the blocking of caspase activity during anoxia and recovery from anoxia. At this point, the possible kinase target of staurosporine in embryos of *A* *. limnaeus* is almost impossible to predict, but future studies that profile gene expression in response to anoxia could help to identify this potentially novel mechanism for regulating caspase activity in response to cellular stress.

### Conclusions

Two independent indicators of cell death/damage indicate a general lack of apoptosis in response to anoxia in embryos of *A* *. limnaeus* . This is especially interesting in light of the large decreases in ATP that are observed during anoxia in this species [12]. Curiously, patterns of apoptosis do not seem to correlate with anoxia tolerance, which suggests that blockage of apoptosis is only one of several mechanisms that support long-term tolerance of anoxia in this species. Reduction of caspase 3/7 activity may be an overall strategy that helps to prevent apoptosis and support the extreme tolerance of anoxia observed in embryos of *A* *. limnaeus* . The mechanism for preventing activation of caspase 3/7 activity in response to anoxia may be dependent on protein kinase activity that is inhibited by staurosporine. Embryos of *A* *. limnaeus* are likely routinely exposed to both short-term and long-term oxygen limitation in their natural developmental environment [29]. Thus, avoidance of anoxia-induced apoptosis during development may be necessary to support normal development and survival in the harsh and unpredictable environment characterized by tropical ephemeral ponds.

### Broader Implications

The ability of *A* *. limnaeus* embryos to endure prolonged anoxia is unique among the vertebrates and provides an opportunity to explore novel mechanisms for supporting survival of anoxia in vertebrate cells. Cells of *A* *. limnaeus* resist activation of necrotic and apoptotic cell death pathways under conditions (no oxygen, low ATP) that would almost certainly lead to cell death in mammalian cells. This study establishes a blockage of apoptotic pathways in anoxic cells that may be kinase dependent, and could lead to novel treatments to block apoptosis in mammalian cells exposed to anoxia.
